# Supplementary material for: Timing of first prenatal ultrasound and associated factors among women who gave birth at health institutions in Ambo Town, central Ethiopia
Source: PLoS One. 2025 Dec 5;20(12):e0338436. doi: 10.1371/journal.pone.0338436 (PMC12680356; doi:10.1371/journal.pone.0338436)
Supplement: S1 File — (PDF) [file pone.0338436.s001.pdf]

English Version Questionnaire

Questionnaire No. [ ] **Date: ______________________________**

Health Institution: ___________________Interviewer Name: ________________________

Instruction: Circle the responses for questions with alternatives and write for open ended.

**Part I: Socio demographic variables**		
**No**	**Questions**	**Response**
1.1	How is your age in completed years?	___________years
1.2	Where is your place of usual residence?	1. Rural 2. Urban
1.3	What is your marital status?	1. Married 2. Single 3. Widowed 4. Divorced
1.4	What is your religion?	1. Orthodox 2. Protestant 3. Muslim 4. Catholic 5. Other specify ___
1.5	What is your ethnicity?	1. Oromo 2. Amhara 3. Tigre 4. Gurage 5. Other specify _______
1.6	What is your educational status?	1. Cannot read and write 2. Able to read and write<br>3. Primary (1-8) 4. Secondary (9-12) 5. College and above
1.7	What is your husband's educational status?	1. Cannot read or write 2. Able to read or write<br>3. Primary (1-8) 4. Secondary (9-12) 5. College and above
1.8	What is your occupation?	1. Housewife 2. Government-Employed<br>3. Self-employed 4. Student 5. Other specify_______
1.9	What is your average monthly income?	___________birr

**Part II: Maternal Antenatal care history**		
2.1	Did you attend ANC during your recent pregnancy?	1. Yes 2. No
2.2	From which health institution did you get antenatal care?	1. Hospital 2. Health center 3. Private clinic

2.3	How many weeks pregnant were you when you had your first antenatal contact during your recent pregnancy?	_______(fill by reviewing participant’s card)
2.4	How many antenatal contacts have you had during your recent pregnancy?	_______contacts
2.5	How many of antenatal contacts were held together with your partner?	_______contacts
**Part III: Maternal Prenatal ultrasound utilization**		
3.1	Have you ever heard of ‘’one ultrasound scan before 24wks of gestation is recommended for pregnant women’’?	1. Yes 2. No , if No skip to Q3.3
3.2	What is your source of information?	1.Health professional 2.Family 3.Media 4.Internet 5.Other specify_____
3.3	How many weeks pregnant were you when you had your first prenatal ultrasound scan during your recent pregnancy?	_______ (fill by reviewing participant’s card)
3.4	Who requested the first prenatal ultrasound during your pregnancy?	1. Yourself 2. Health professional 3. Other specify _______
3.5	The number of times you had prenatal ultrasound scans during recent pregnancy?	___________ times
3.6	Have you used prenatal ultrasound scan during your previous pregnancies?	1. Yes 2. No
3.7	How much time do you wait to get a prenatal ultrasound service?	___________min
3.8	Have you got an explanation about the image result of a prenatal ultrasound scan?	1. Yes 2. No
**Part IV: Maternal Health related factors**		
4.1	Is your recent pregnancy intended?	1. Yes 2. No
4.2	Do you have a known health problem before pregnancy?	1. Yes 2. No, if yes what___________
4.3	Have you faced a health problem during your recent pregnancy?	1. Yes 2. No , if No skip to Q4.5
4.4	What kind of health problem did you face?<br>(multiple response is possible)	1. Antepartum hemorrhage<br>2. Postpartum hemorrhage

		3. Preterm labour<br>4. Gestational hypertension<br>5. Gestational diabetes<br>6. Premature rupture of membrane<br>7. Intrauterine fetal death<br>8. Other specify___________
4.5	Have you been drinking alcohol during your recent pregnancy?	1. Yes 2. No
**Part V: Birth related factors**		
5.1	Where was your recent pregnancy place of delivery?	1. Hospital 2. Health center 3. Other specify_______
5.2	Recent pregnancy mode of delivery?	1. Vaginal delivery 2. Caesarian delivery
5.3	Do you have a past history of Caesarian delivery?	1. Yes 2. No
5.4	What was the outcome of your recent pregnancy?	1. Alive birth 2. Still birth 3. Other specify_______
5.5	How many babies have you delivered?	1. Singleton 2. Twins 3. Other specify _______
5.6	Gestational age at birth?	_______ (fill by reviewing participant's card)
5.7	Birth weight?	_______ (fill by reviewing participant's card)
5.8	Is the baby admitted to neonatal intensive care unit after birth?	1. Yes 2. No
**Part VI: Reproductive factors**		
6.1	Gravidity?	___________
6.2	Parity?	___________
6.3	Have you ever had bad obstetric history?	1. Yes 2. No, if No skip to Q7.1
6.4	Have you ever had a history of abortion?	1. Yes 2. No
6.5	Have you ever had a history of premature birth?	1. Yes 2. No
6.6	Have you ever had a history of congenital anomaly?	1. Yes 2. No

6.7	Have you ever had any other abnormality?	______________________________

Part VII: Maternal Knowledge of prenatal ultrasound		Yes	No	I don't know
7.1	Prenatal ultrasound confirm pregnancy			
7.2	Prenatal ultrasound determine the sex of the baby			
7.3	Prenatal ultrasound determine the fetal position			
7.4	Prenatal ultrasound determine the placenta position			
7.5	Prenatal ultrasound determine the expected date of delivery			
7.6	Prenatal ultrasound detect congenital abnormalities			
7.7	Prenatal ultrasound detect ectopic pregnancy			
7.8	Prenatal ultrasound measure amniotic fluid volume			
7.9	Prenatal ultrasound assess well-being of the fetus			
7.10	Prenatal ultrasound confirm the presence of multiple pregnancies			
7.11	Prenatal ultrasound estimate fetal weight			
7.12	Prenatal ultrasound estimate gestational age			

Part VIII: Maternal attitude towards prenatal ultrasound		1	2	3	4	5
8.1	Prenatal ultrasound is safe for mother					
8.2	Prenatal ultrasound is safe for fetus					
8.3	Prenatal ultrasound can lead to congenital anomaly					
8.4	Prenatal ultrasound in an essential investigation during pregnancy					
8.5	Knowing the sex of your child before birth will bring bad luck					
8.6	Terminate the pregnancy if the sex of the child is other than you prefer					
8.7	Educating others about prenatal ultrasound is important					
8.8	Prenatal ultrasound can lead to cancer					
8.9	Prenatal ultrasound give accurate information					
8.10	Prenatal ultrasound tends to be offered by chance					

Note: 1. Strongly agree 2. Agree 3. Neutral 4. Disagree 5. Strongly disagree

Thank you!
