# Supplementary material for: Timing of first prenatal ultrasound and associated factors among women who gave birth at health institutions in Ambo Town, central Ethiopia
Source: PLoS One. 2025 Dec 5;20(12):e0338436. doi: 10.1371/journal.pone.0338436 (PMC12680356; doi:10.1371/journal.pone.0338436)
Supplement: Attachment — Submitted filename: Comments by Peninah Agaba.docx [file pone.0338436.s003.docx]

Comments by Peninah Agaba

**Manuscript title: Timing of first prenatal Ultrasound and associated factors among women who gave birth at health institutions in Ambo Town, central Ethiopia.**

**Comments**

There are many typos and grammatical errors in the paper which should be corrected as soon as possible.

**Abstract**

Methods: Is the data source health facility records or exit interviews at the health facility or a mix of both. This should be made clear

**Results:** Why not say ‘Overall, 71%....

**Conclusion:** women didn’t had is a wrong tense

**Background:**

Paragraph 5, is the relationship between US and those factors confirmed or it could be due to other government campaigns. Use a word that shows the likely relationship

Methods: in the abstract, you say participants were systematically selected while in the methods section, you say they were randomly selected. Which one is which?

The exclusion criteria is not right because this binary analysis should include yes and no. Include both in the analysis

How many days postpartum were these women? Were they interviewed from the ward or they had already been discharged and you found them at their homes?

Attach a copy of the questionnaire

**Sample size & sampling strategy**

The sample size determination write-up is not clear e.g what is reference 17 after 402 for? What is initial sample size? The word ‘of’ is missing in that sentence of initial sample size.

The paragraph on sampling strategy is not clear. Present it in a clear & concise way. It is confusing the way it is. If you used records of July to September to get proportions, to guide the sample selection in each health facility, let it be clear. Which proportions were they that guided the samples in each health centres. Use percentages to give a picture of the proportions than absolute numbers- proportions of births & proportions for sample distribution.

The selection processes of participants must be described succinctly

I would like to know how this study becomes cross-sectional

In the study area, you talked about several health facilities, how did you zero down on the three health facilities

How many respondents for the pre-test? Were they different from the ones included in the final sample or you included pre-test data in the final analysis?

Dis you use paper based questionnaires or computer assisted personal interviews?

What other independent variables were included in the model?

**Results**

**Descriptive results**

The first sentence has a wrong tense- the word were is misplaced

On average income, remove brackets because the figures are part of the sentence otherwise, the sentence is incomplete

‘Had an information’, ‘received from health professional’ these are not right, re-write them

Were delivered in the hospitals or delivered in the hospital

Were weighting????

**Factors associated with timing of first prenatal ultrasound**

To timely use ultra sound scan ot to have a timely ultra sound scan??

Check your brackets as you report the AOR and CIs

Could you briefly talk about the bivariate results? Mentioning them where you have is confusing as the reader expects multivariate factors & then you begin with bivariate factors.

I think the variable should be previous history of prenatal ultrasound

The referencing style seems to be having issues, why do you have two brackets with different references in one place. You need to leave a space befor the brackets throughout the document
