# Supplementary material for: Timing of first prenatal ultrasound and associated factors among women who gave birth at health institutions in Ambo Town, central Ethiopia
Source: PLoS One. 2025 Dec 5;20(12):e0338436. doi: 10.1371/journal.pone.0338436 (PMC12680356; doi:10.1371/journal.pone.0338436)
Supplement: Attachment — Submitted filename: R1 Response to Reviewers.docx [file pone.0338436.s004.docx]

**PONE-D-25-08139**

**Timing of first prenatal Ultrasound and associated factors among women who gave birth at health institutions in Ambo Town, central Ethiopia.**

**PLOS ONE**

**To: Editor of PLOS ONE Journal**

**Dear Sir/Madam,**

We wish to remind you of our submission of the manuscript entitled “*Timing of First Prenatal Ultrasound and Factors Associated with It Among Women Who Gave Birth at Health Institutions in Ambo Town, Central Ethiopia*” for publication consideration in PLOS ONE. We express our gratitude for your efforts in reviewing our article. In accordance with the feedback provided by the reviewers and editor, we have revised and modified the manuscript, addressing each comment individually as detailed below. The revised version, with tracked changes and clean version are attached for your reference. The responses to the reviewers' and editor's comments are outlined below.

**Gizachew Abdissa Bulto**

**Email:** [**gizachab@yahoo.com**](mailto:gizachab@yahoo.com)

**Corresponding Author**

**Editor Comments**:

Thank you for submitting your manuscript to PLOS ONE. After careful consideration, we feel that it has merit but does not fully meet PLOS ONE’s publication criteria as it currently stands. Therefore, we invite you to submit a revised version of the manuscript that addresses the points raised during the review process.
**Author’s response**: We are grateful for your valuable comments, suggestions to enrich our paper. Thank you again for considering our article for revision.
Please submit your revised manuscript by Sep 11 2025 11:59PM. If you will need more time than this to complete your revisions, please reply to this message or contact the journal office at [plosone@plos.org](mailto:plosone@plos.org). When you're ready to submit your revision, log on to https://www.editorialmanager.com/pone/ and select the 'Submissions Needing Revision' folder to locate your manuscript file.
Please include the following items when submitting your revised manuscript:

- A rebuttal letter that responds to each point raised by the academic editor and reviewer(s). You should upload this letter as a separate file labeled 'Response to Reviewers'.
- A marked-up copy of your manuscript that highlights changes made to the original version. You should upload this as a separate file labeled 'Revised Manuscript with Track Changes'.
- An unmarked version of your revised paper without tracked changes. You should upload this as a separate file labeled 'Manuscript'.

**Author’s response**: Thank you! We did it accordingly.

If you would like to make changes to your financial disclosure, please include your updated statement in your cover letter. Guidelines for resubmitting your figure files are available below the reviewer comments at the end of this letter.
**Author’s response**: Thank you. No change has been made.
If applicable, we recommend that you deposit your laboratory protocols in protocols.io to enhance the reproducibility of your results. Protocols.io assigns your protocol its own identifier (DOI) so that it can be cited independently in the future. For instructions see: [https://journals.plos.org/plosone/s/submission-guidelines#loc-laboratory-protocols](https://track.editorialmanager.com/CL0/https:%2F%2Fjournals.plos.org%2Fplosone%2Fs%2Fsubmission-guidelines%23loc-laboratory-protocols/1/010f0198661230ee-2bef11d0-52c5-4fc7-9fca-6d0abab0d947-000000/IBWoxjbUy364A1D4h3E4-IRnBvRPFgMgwKprwFCDSYA=222). Additionally, PLOS ONE offers an option for publishing peer-reviewed Lab Protocol articles, which describe protocols hosted on protocols.io. Read more information on sharing protocols at [https://plos.org/protocols?utm_medium=editorial-email&utm_source=authorletters&utm_campaign=protocols](https://track.editorialmanager.com/CL0/https:%2F%2Fplos.org%2Fprotocols%3Futm_medium=editorial-email%26utm_source=authorletters%26utm_campaign=protocols/1/010f0198661230ee-2bef11d0-52c5-4fc7-9fca-6d0abab0d947-000000/0huo2JvUfPp6uGKSLOfN86t2w7uFu0xX4eDqq74enCU=222).

Academic Editor
**Author’s response**: Thank you!

**Journal Requirements:**

When submitting your revision, we need you to address these additional requirements.

1. Please ensure that your manuscript meets PLOS ONE's style requirements, including those for file naming. The PLOS ONE style templates can be found at

https://journals.plos.org/plosone/s/file?id=wjVg/PLOSOne_formatting_sample_main_body.pdf and 
https://journals.plos.org/plosone/s/file?id=ba62/PLOSOne_formatting_sample_title_authors_affiliations.pdf

2. Please note that funding information should not appear in the Acknowledgments section or other areas of your manuscript. We will only publish funding information present in the Funding Statement section of the online submission form. Please remove any funding-related text from the manuscript.

3. In the online submission form, you indicated that:

“The datasets used and/or analyzed during the current study are available in the manuscript and details from the corresponding author on reasonable request.”

All PLOS journals now require all data underlying the findings described in their manuscript to be freely available to other researchers, either 1. In a public repository, 2. Within the manuscript itself, or 3. Uploaded as supplementary information.

This policy applies to all data except where public deposition would breach compliance with the protocol approved by your research ethics board. If your data cannot be made publicly available for ethical or legal reasons (e.g., public availability would compromise patient privacy), please explain your reasons on resubmission and your exemption request will be escalated for approval.

**Authors’ response**: Thank you! We have revised the data availability statement in to: “*All relevant data are within the paper and its Supporting Information files*.”

4. Please include a separate caption for each figure in your manuscript.

**Author’s response**: Thank you, we have included in the revised version.

5. If the reviewer comments include a recommendation to cite specific previously published works, please review and evaluate these publications to determine whether they are relevant and should be cited. There is no requirement to cite these works unless the editor has indicated otherwise. 
**Author’s response**: Thank you, we appreciated!

**Reviewers' comments:
Reviewer's Responses to Questions**

**Comments to the Author**
1. Is the manuscript technically sound, and do the data support the conclusions?

The manuscript must describe a technically sound piece of scientific research with data that supports the conclusions. Experiments must have been conducted rigorously, with appropriate controls, replication, and sample sizes. The conclusions must be drawn appropriately based on the data presented.

Reviewer #1: Yes

Reviewer #2: Yes

Reviewer #3: Yes

Reviewer #4: Yes

Reviewer #5: Yes

Reviewer #6: Partly

2. Has the statistical analysis been performed appropriately and rigorously?

 Reviewer #1: Yes

Reviewer #2: Yes

Reviewer #3: Yes

Reviewer #4: Yes

Reviewer #5: Yes

Reviewer #6: Yes

**Author’s response**: Thank you, we appreciated it!

 3. Have the authors made all data underlying the findings in their manuscript fully available?

The [PLOS Data policy](http://track.editorialmanager.com/CL0/http:%2F%2Fwww.plosone.org%2Fstatic%2Fpolicies.action%23sharing/1/010f0198661230ee-2bef11d0-52c5-4fc7-9fca-6d0abab0d947-000000/HCxZXwVwlnLNFtUIK1PGyMGQMWF0i2tJLXDCW2QFnGA=222) requires authors to make all data underlying the findings described in their manuscript fully available without restriction, with rare exception (please refer to the Data Availability Statement in the manuscript PDF file). The data should be provided as part of the manuscript or its supporting information, or deposited to a public repository. For example, in addition to summary statistics, the data points behind means, medians and variance measures should be available. If there are restrictions on publicly sharing data—e.g. participant privacy or use of data from a third party—those must be specified.

 Reviewer #1: Yes

Reviewer #2: Yes

Reviewer #3: Yes

Reviewer #4: Yes

Reviewer #5: Yes

Reviewer #6: No

 Authors response: Thank you, we have included on the revised paper.

4. Is the manuscript presented in an intelligible fashion and written in standard English?

PLOS ONE does not copyedit accepted manuscripts, so the language in submitted articles must be clear, correct, and unambiguous. Any typographical or grammatical errors should be corrected at revision, so please note any specific errors here.

Reviewer #1: Yes

Reviewer #2: Yes

Reviewer #3: Yes

Reviewer #4: Yes

Reviewer #5: Yes

Reviewer #6: Yes

**Author’s response**: Thank you!

5. Review Comments to the Author

Please use the space provided to explain your answers to the questions above. You may also include additional comments for the author, including concerns about dual publication, research ethics, or publication ethics. (Please upload your review as an attachment if it exceeds 20,000 characters)

**Reviewer #1:**

The paper is well-written and offers valuable insights into the existing body of knowledge; however, I have some concerns that require attention.
1. In the abstract's background section, it is better to change the statement “Hence, it was aimed to assess…” with “Hence, the study aimed to assess…”

**Authors response**: Thank you we have revised it accordingly.
2. Similarly in the abstract's result section write the variable as “living in urban area”

**Authors response**: We accepted your comment and incorporated in the revised version.
3. Again, in the abstract's conclusion section, change the term “place of residence” to “urban residence” to make them consistent.

**Authors response**: We accepted your comment and incorporated in the revised version.
4. In the background, you repeated this statement, “Prenatal ultrasound is an essential core package of routine Antenatal care (ANC)“, two times.

**Authors response**: It was a nice observation. Thank you we have corrected it in the revised version removing the later statement.
5. Again in the background why you use the term furthermore in “Furthermore, the introduction…?

**Authors response**: Thank you we have omitted the word from the third paragraph of the introduction. Now revised in to: *The introduction of a timely prenatal ultrasound service at public hospitals in Ethiopia increases* …..
6. In the method part, it is better to change the sentence “All women who delivered at public health facilities in Ambo town and randomly selected were the study population.” with "All randomly selected women who delivered at public health facilities in Ambo town were the study population."

**Authors response**: Thank you for your suggestion, we accepted your comment and revised accordingly.
7. You use the mean and standard deviation as summary measures in all documents. Did you check your data distribution? You used the mean value as a cut-off point to categorise the knowledge and attitude. Did you check your data distribution for these variables?

Thank **Authors response**: you for this valid concern. We have checked the data for normality distribution and it was found a normally distributed. We have included the statement which support this point under the ‘Data processing and analysis’ section as: “*The normality of the distribution concerning participants' knowledge and attitudes towards perinatal ultrasound was assessed through visual inspection of histograms and Q–Q plots. The findings demonstrated that the data were normally distributed*.”
8. Add references for the information you use in the study area and merge some unmerged references like in the background, method… Check the whole document.

**Authors response**: Thank you very much for your suggestions we revised it through merging the references under the introduction, methods and discussion sections accordingly. Additionally, we added one reference for the information under the study area.
9. How did you handle cases where a woman received antenatal care (ANC) at another health facility and delivered at one of the study facilities during your study period?

**Authors response**: Thank you for raising this valid concern! If the women clearly recall or have documented evidence of their timing of first ultrasound scan they were included, Otherwise, excluded. We added statement regarding this as: “*Women who did not undergo a prenatal ultrasound scan or who could not recall or had no record of their first ultrasound scan during their recent pregnancy were excluded*.”
10. Is assessing women’s knowledge and attitude toward prenatal ultrasound at the time of delivery not influenced by the kind of care they received throughout their pregnancy, as all women had a history of ANC? Could you please explain why the study was not conducted while they were receiving ANC?

**Authors’ response:**
Thank you for raising this concern. We acknowledge that women’s knowledge and attitudes toward prenatal ultrasound could potentially be influenced by the type of care they received and the level of health facility they attended during pregnancy. In our study, we collected information on the number of ANC contacts, facility-related characteristics, and other relevant variables; however, none of these were found to significantly affect the timing of the first ultrasound.

While it would have been possible to assess timing among women during their pregnancy, our primary objective was to examine the timing and frequency of the first prenatal ultrasound in relation to pregnancy outcomes, including birth outcomes. For this reason, data were collected from women after delivery. Although our ability to compare outcomes across categories was limited by small sample sizes for each categories, we found no meaningful differences in the outcomes assessed.

**Reviewer #2:**

This study employed a health-facility-based cross-sectional design to assess the timing of first prenatal ultrasound and associated factors in Ambo Town, central Ethiopia. While the methodology has several strengths, some aspects needs critical consideration.

**Authors response**: Thank you very much we appreciate your valuable feedbacks.

Rationale:
The introduction did not contextualize why Ambo Town was selected for this study (e.g., urban-rural disparities in healthcare access) and clarify if prior local data existed on this topic.

**Authors response**: Thank you! This study was conducted with limited budget as a fulfillment for Master’s degree and could not cover a wider geographical area. But we believe we had also included women from rural areas (20.4%) who came to give birth at those Public Health facilities in Ambo town which includes General and Teaching Hospitals providing services for more than 3 million populations. We have included one statement as a rationale for conducting this study in the current study area. Which reads as: “*Whilst national guidelines recommend early prenatal ultrasound (prior to 24 weeks of gestational age), there is a dearth of information regarding its implementation in Ethiopian public health facilities. Furthermore, no data are available on this topic in the study area to guide local interventions. ..*.”

Study Design and Setting
This study covers a relatively short period (September 12 to October 30, 2022) and captures a snapshot, however it might not account for seasonal variations or other temporal factors that could influence ultrasound utilization. (could be addressed in limitation)

**Authors response**: Thank you for raising this concern. Although there might be seasonal variations in the utilization of ANC and timing of ultrasound use among women, in the current study we collected data from women who gave birth at public health facilities and we collected data for about seven weeks which could touch three seasons with additional more than a month in the other season. Hence their ultrasound use and timing issue could be addressed within the 10 months duration. It is true that cross sectional study gives us a snapshot of information. We have already included a limitation as: “*Additionally, since it only included women who gave birth at public health facilities in Ambo town, the findings may not be generalizable to women who delivered at home.*”

Tool Development:
Since the questionnaire were self-developed, how was the construct validity ensured? How was it considered enough to assess the knowledge and attitude? This study talks about language validity and internal consistency for intended constructs but not mentioned regarding how many items would make eligible construct for the knowledge and attitude. (explain the method)
BIAS and confounders addressing:

**Authors response**: Thank you. We adapted the questionnaire from the previously conducted related literatures. We have checked the internal reliability of the tool with Cronbach alpha test and we found: the Cronbach's alpha value was calculated at 0.87, and 0.85 for attitude assessment items. We have already indicated the number of items included for determining the knowledge status and for the attitude score under the operational definitions. The statements read as:

“*Knowledge on prenatal ultrasound: Twelve questions were used to assess participants' knowledge of prenatal ultrasound. The average score was to determine the status. Overall knowledge status was classified as 'good' if a participant's score was equal to or exceeded the mean, and 'poor' if it fell below the mean score of these knowledge-assessing items (15).*

*The attitude score was determined based on a composite value derived from responses to a Likert-type scale. The overall attitude was determined by computing the average of all ten attitude-related questions. Respondents whose scores were equal to or exceeded the mean value were classified as having a favorable attitude, whilst those scoring below the mean were deemed to have an unfavorable attitude towards prenatal ultrasound (15).”*

Exclusion Criteria: Women who had no prenatal ultrasound scan at all during their recent pregnancy were excluded. While this focuses the study on the timing of first ultrasound, it means the study does not capture the portion of the population that does not receive any ultrasound, which could be a significant public health concern. Selection bias.

**Authors response**: Thank you for raising this concern! We acknowledge that excluding women who did not undergo any ultrasound scan may omit a population of high public health significance. According to WHO, the Ethiopian national guidelines, and other international recommendations, every pregnant woman should receive an early ultrasound scan before 24 weeks of gestation. However, there are indeed women who do not have any ultrasound during pregnancy.

The objective of our current study was specifically to assess the timing of the first prenatal ultrasound among women who had at least one scan during their most recent pregnancy. While determining the proportion of women who never had an ultrasound would also be valuable, it would not be possible to analyze the timing for those without a scan along with those who had it. For this reason, we excluded women with no ultrasound from our analysis.

Unadjusted variables like healthcare provider density or facility resources (ultrasound availability) could influence results. How was this addressed? (potential confounders)

**Authors response**: Thank you for raising this concern! It would be good to triangulate the current finding through exploring the health care providers and health facilities related factors that would possibly affect the ultrasound scan and its timing. We appreciate your comment and it would be good if the study addressed those possible factors. Therefore, we included a statement under the limitation which reads: *“… The current study doesn’t take into account the health facilities and care providers related factors which could be important to comprehensively explore the factors which could affect perinatal ultrasound scan and its timing. These were the main limitations of the study*.” We have also included under the recommendation section for researchers.

Facility-Based Study Design: The findings may not be applicable to women who delivered at home or in private health facilities creating selection bias. Is this a limitation? Because generally, the percentage of women delivering in private facility might be much higher than home delivery. Clarify the local delivery practices to give the reader a clear picture.

**Authors response**: Thank you! Yes, it would affect but in the current study area or Ambo town there is no private health facilities which were providing the delivery service.

We have also added a statement under the study design, area and period, which explain as only the public health facilities were providing delivery services. Which reads as: “*Only Amo University Referral Hospital, Ambo General Hospital, Ambo and Awaro Health centers were providing delivery services in the town and were selected for the current study.”*

We have also included one statement as a limitation as the current study only considered those women who gave birth at health facilities.

*The statement reads as: “Additionally, since it only included women who gave birth at public health facilities in Ambo town, the findings may not be generalizable to women who delivered at home.”*

Recall Bias in Reporting of Ultrasound Timing
The study tried to minimize the recall bias by cross checking with record card, however, the study did not explicitly report on discrepancies found between interview responses and medical records and how it was resolved regarding ultrasound timing.
**Authors response**: Thank you very much. We have included on the revised manuscript. Included a statement which state how the timing of the ultrasound scan were recorded which read as:

“… *The timing of the first ultrasound scan was determined using both the printed scan results and the one recorded by healthcare providers in the participants’ medical records. …”*
Discussion:
The study overstates causality while explaining implications, such as "good knowledge" leads to timely scans” in absence of longitudinal data.
The study does not explore why health centers underperform for e.g., lack of machines, trained staff etc.

**Authors response**: Thank you. We reported having good knowledge as one factor which was found to be significantly associated with the early ultrasound scan. We acknowledged the issues related to health facilities and providers related factors as a limitation.

Review
This study employed a health-facility-based cross-sectional design to assess the timing of first prenatal ultrasound and associated factors among women who delivered in Ambo Town, central Ethiopia. While the methodology demonstrates several strengths, such as systematic random sampling, robust sample size calculation, and use of validated tools, certain aspects needs some consideration to enhance rigor and generalizability.

**Authors response**: Thank you these seems repeated here again. We have addressed it above.

Rationale: The introduction effectively establishes the importance of timely prenatal ultrasound per WHO and Ethiopian guidelines. However, it does not explicitly justify the selection of Ambo Town, particularly regarding urban-rural disparities in healthcare access or whether prior local studies on this topic exist. Clarifying these points would strengthen the study’s relevance to regional policy and practice.

**Authors response**: We have again addressed this concern and comment above.

Study Design: The data collection period (September 12 to October 30, 2022) provides a snapshot of ultrasound utilization but may not account for seasonal variations (e.g., agricultural cycles affecting healthcare access) or other temporal factors. This is not acknowledged in the limitation.
Tool Development and Validity
The study used a self-developed questionnaire, with translation and back-translation ensuring language validity and Cronbach’s alpha (0.85–0.87) confirming internal consistency. However, the manuscript does not detail how construct validity was established (e.g., expert review, factor analysis) or justify the sufficiency of the 12-item knowledge scale and 10-item attitude scale. A clearer description of item selection and validation methods would add up to the validity and reliability of the tool.

**Authors response**: we have also addressed this issue above. It was repeated here. *We have included statement on the content and face validity of the tool.*
Selection Bias:
Exclusion of Women Without Ultrasound: The study excluded women who had no prenatal ultrasound, omitting a critical population segment that may face systemic barriers (e.g., poverty, distance). This introduces selection bias and limits understanding of non-utilization drivers.
Facility-Based Design: Findings may not generalize to women delivering at home or in private facilities. The manuscript should clarify local delivery practices (e.g., percentage of home births) to contextualize this limitation.
**Authors response**: Thank you we have addressed these concerns above.

Unmeasured Confounders: Facility-level factors (e.g., ultrasound machine availability, staff training) were not adjusted for, potentially confounding associations (e.g., hospital ANC attendance vs. health centers). Acknowledging these unmeasured confounders in limitation would shed light on scope of the utility of the findings.
Recall Bias: While the study cross-checked self-reported ultrasound timing with medical records, it did not report discrepancies or resolution methods. Quantifying discordance rates would strengthen validity claims.

**Authors response**: It was repeated here from the comment raised above. We have already addressed these comments.
Discussion: The discussion appropriately compares findings with global and regional studies but overstates causal relationships (e.g., "good knowledge leads to timely scans") without longitudinal or qualitative data. Additionally, it does not explore structural barriers (e.g., health center resource gaps) that may explain underperformance. Addressing these gaps would provide actionable insights for policymakers.

**Authors response**: We acknowledged it under the limitation section. Thank you!

**Reviewer #3:**

I had gone through “Timing of first prenatal Ultrasound and associated factors among women who gave birth at health institutions in Ambo Town, central Ethiopia.” Few minor modifications need to be done
1. Title can be modified as ‘Timing of first prenatal Ultrasound and factors associated with it among women who gave birth at health institutions in Ambo Town, central Ethiopia.’

**Author’s response**: We thank you for your suggestion. We have modified it accordingly.

2. In result part the author had mentioned about “The line graph below shows the proportion of mothers who had their first prenatal ultrasound scan along with the new 8 contact ANC model (Fig. 1). It would be better if the author give an introduction to new 8 contact ANC model in the background, so that reviewers or readers understand it well. The title of Fig 1 is confusing can be modified. The figure shows the proportion of women taking first ultrasound in various weeks of gestation.

**Author’s response**: Thank you for your comment. We have accepted and revised it. We just omitted the new ANC contact model from the title and text. The revision reads as:

*“The figure shows the proportion of women who had their first prenatal ultrasound scan along with their corresponding gestational age across the 8 contacts ANC (Fig. 1).”*

*The title is also revised into: Figure 1: Timing of the First Prenatal Ultrasound Scan Among Women Who Gave Birth in Public Health Institutions, Ambo Town, Ethiopia, 2022 (N = 442).”*

3. In knowledge part one of the question is to determine the sex of the baby. Usually in India sex determination through prenatal USG is prohibited or punishable act. I don’t know about the rules and regulation of Ethiopia Government.

**Author’s response**: Thank you for raising this concern. In Ethiopia, reporting the sex of the fetus is not prohibited. If a woman requests this information, the provider will disclose it. In fact, it is common for women to request fetal sex determination, and many even consider it a routine component of the ultrasound report.

**Reviewer #4:**

This manuscript addresses an important aspect of maternal health services—timely utilization of prenatal ultrasound and identifies associated factors within the Ethiopian context. The study is relevant and aligns with both national and global efforts to reduce maternal and neonatal morbidity and mortality. However, several points require improvement and refinement as follows:
1. Novelty and Scientific Contribution
The authors should clarify the novelty of this study compared to previous studies conducted in other regions of Ethiopia (such as Jimma) or other countries. What makes this study distinct, and how do the findings contribute to strengthening local or regional health policies? Additionally, the authors are encouraged to justify why Ambo Town was chosen as the study location. Is it due to suboptimal ANC services or high maternal mortality in the area?

**Author’s response**: Dear reviewer we thank you for your valuable comments. We have included in the revision under the introduction section. The study area was selected purposively for its proximity to where the university is located and due to the limited available resources. The last paragraph of the introduction reads as:

*“The previous studies only assessed the proportion of women who had ultrasound scan among pregnant women, not the timing of their first perinatal ultrasound. Furthermore, there is no data available on this topic in the study area to guide local interventions. Consequently, the researchers conducted this study to determine the timing of initial prenatal ultrasound scans and the associated factors among women who gave birth in public health institutions in Ambo town, Ethiopia, in 2022.”*

2. Inconsistency Between p-value and Confidence Interval (CI)
In the multivariate analysis, the variable “previous history of prenatal ultrasound” shows an Adjusted Odds Ratio (AOR) of 1.20 with a p-value of 0.009, but the confidence interval (CI: 0.69–2.08) includes the value 1. This is statistically inconsistent because an effect should not be considered significant if the CI includes 1. The authors are requested to verify and correct the data or provide a clear explanation for this discrepancy.

**Author’s response**: Thank you. It was an editorial problem, while writing the manuscript. We have computed again and corrected it on the revised version of the article. Now corrected to AOR= 0.51, 95% CI: 0.12-2.18) and P-value= 0.371)

3. Attitude as a Non-significant Factor
Although the assessment of attitudes toward prenatal ultrasound is presented, the results indicate that attitude was not significantly associated with the timely use of ultrasound. The authors should discuss why this may be the case and consider the implications for community health education strategies.

**Author’s response**: Thank you for your suggestions. We have included one paragraph discussion for attitude. The paragraph read as:

*“The results revealed that attitude was not found significantly associated with the early use of ultrasound among women in the multivariable model. This could be attributed to the fact that nearly half of the participants held an unfavorable attitude towards prenatal ultrasound, and some rumors about its potential effects on the fetus continue to circulate among women. Additionally, structural and health system barriers such as the late initiation of antenatal care and the limited availability of ultrasound services may impede early uptake regardless of attitude. Consequently, this finding highlights the necessity of complementing attitude-focused messaging with targeted interventions that address these practical barriers and emphasize the importance of early attendance for antenatal care and having early prenatal ultrasound (15,28,29).”
4. Study Limitations
Although limitations are briefly mentioned, they should be expanded, particularly regarding potential selection bias (since the study only included women who gave birth in public health facilities) and possible recall bias, even though medical records were also used.*

**Author’s response**: Thank you very much we have added more limitations of the current study under the limitation section. The limitation now reads as:

“*The study used a cross-sectional design, which makes it difficult to establish causality. Additionally, since it only included women who gave birth at public health facilities in Ambo town, the findings may not be generalizable to women who delivered at home. Recall bias might be introduced for some of the variables which require the women to remember to past events to respond. The current study doesn’t take into account the health facilities and care providers related factors which could be important to comprehensively explore the factors which could affect perinatal ultrasound scan and its timing. These were the main limitations of the study*.”

**Reviewer #5:**

Thank you for writing this interesting paper on an important issue. I enjoyed reading it. One of the most important benefits of ultrasound regarding ascertainment of the expected date of delivery is the avoidance of pregnancies that proceed past 41 weeks, as these prolonged pregnancies carry risks of stillbirth and other complications such as shoulder dystocia associated with large birthweight. I feel that you should mention this issue of avoidance of prolonged pregnancy by ascertainment of dates in pregnancies where women are not aware of their dates. Also perhaps you could enhance the discussion by considering more about the reasons why dates may be so uncertain, for example, breastfeeding a previous baby, certain contraceptives etc.

**Author’s response**: Thank you for your suggestions. We have incorporated your suggestion under introduction section in the revised version. The revised version reads as: “… *This unreliable estimation of gestational age can pose challenges when managing conditions such as preterm labor and intrauterine growth restriction and induction of labor for post term pregnancy (10). Studies also reported the use of early ultrasound scan to determine gestational age lowered the rate of induction for postdate pregnancy (20)(21).”*

It would also be pertinent to discuss the financial aspects of US. Who pays for scans? Is it out of pocket? If so this may be a major reason for not being able to attend for a scan.

**Author’s response**: Here in Ethiopia the public health facilities are providing free services for women during pregnancy, childbirth and postpartum periods. They don’t pay for ultrasound service as well.

What happens when anomalies are detected on US? Is there an option to undergo safe abortion if significant anomalies are detected? This is another issue that may affect a woman's choice to attend for US.

**Author’s response**: Thank you! Yes, termination of pregnancy is permitted for those who are found to have incurable anomalies or for those who couldn’t be compatible with life after birth.

In the introduction you talk about problems being "tackled". This is a rather strange word to use in this context and maybe you could find an alternative?

**Author’s response**: It was a nice observation. We have revised the whole statement which now read. “*These conditions could potentially be addressed if all women received a timely prenatal ultrasound.*”

Otherwise there are some minor grammatical errors but these do not affect the readability or comprehension of the paper which is f=good overall.

**Author’s response**: We appreciate your valuable comments and suggestions.

**Reviewer #6:**

This paper addresses a significant public health issue concerning Ethiopian women, highlighting the importance of prenatal ultrasound as a tool for both prenatal diagnosis and safe motherhood. However, several critical limitations must be addressed.
**Author’s response**: Thank you for your kind words and comments.

The most substantial concern is the absence of a coherent conceptual framework. In the Introduction, the author emphasizes the role of early antenatal ultrasound in improving perinatal outcomes and reducing maternal mortality. Yet, there is no discussion of the structural barriers limiting access to this service in the region—arguably the central focus of the study.
**Author’s response**: Thank you for raising this concern. We acknowledge it, if the structural and other provider related factors which could potentially affect the women from having early ultrasound scan could be addressed to comprehensively understand the issue. In our case we acknowledged this a limitation of the current study under our limitation section. We added a statement which reads:

“… *The current study doesn’t take into account the health facilities and care providers related factors which could be important to comprehensively explore the factors which could affect perinatal ultrasound scan and its timing..*.”

Methodologically, the study adopts a cross-sectional design across multiple centers in the Ambo region but inexplicably excludes women who did not undergo antenatal ultrasound. More problematically, the sample size calculation employs a double proportion population formula, using a history of previous abortion as the "risk factor" to compare exposed and unexposed cases. Only later do the authors introduce "knowledge of prenatal ultrasound" as a variable worth examining among other potential factors influencing the timing of the first prenatal scan.

***Author’s response****: Thank you! We appreciate your comment. We have calculated sample size using both single population proportion formula and double population proportion using the Epi Info software. The sample size calculated for the proportion of early ultrasound was found to be 422 and for the factors associated which was 442. Hence we took the largest one from the two just to briefly indicate it. If required we can share the details on how we calculated our sample size.*

*Moreover, we acknowledge that excluding women who did not undergo any ultrasound scan may omit a population of high public health significance. According to WHO, the Ethiopian national guidelines, and other international recommendations, every pregnant woman should receive an early ultrasound scan before 24 weeks of gestation. However, there are indeed women who do not have any ultrasound during pregnancy. The objective of our current study was specifically to assess the timing of the first prenatal ultrasound among women who had at least one scan during their most recent pregnancy. While determining the proportion of women who never had an ultrasound would also be valuable, it would not be possible to analyze the timing for those without a scan along with those who had it. For this reason, we excluded women with no ultrasound from our analysis.*

In essence, there is a disconnect between the introduction, sample size justification, and variable selection. This inconsistency has a cascading effect on the results, where the timing of the first prenatal ultrasound abruptly becomes the outcome variable—apparently the true research question—while a multivariate analysis is conducted on demographic and socioeconomic variables that were neither theoretically grounded nor operationalized (with the exception of prior ultrasound knowledge). Given these fundamental methodological flaws, a detailed discussion of the results is unwarranted. However, several stylistic issues should be corrected:

**Author’s response**: Thank you. We have tried to revise the whole document incorporating the whole reviewers’ comment and we hope it was significantly improved on the revised version. On the sample size we used the variable which gave us the maximum sample among the factors from the previous study. We are happy to put any variable which you think better added or required to be put under operational definition in the future revision. We also believe majority of the variables were from previously conducted studies.

We are happy to address if additional concern from your end which you think must be addressed or revised.

Numerical presentation: If numbers and percentages are already provided in parentheses, spelling out the number is redundant (e.g., "262 (59.3%)" suffices; "two hundred sixty-two (59.3%)" is unnecessary).
**Author’s response**: Thank you for your comment. We have revised it accordingly in the whole document. The statement now reads as: “… *A total of 262 respondents (59.3%) were multigravida. Adverse obstetric history was reported by 39 respondents (8.9%), and 30 respondents (6.8%) had a history of abortion. Furthermore, 382 mothers (86.4%) delivered in hospitals, with 120 of them (27.1%) undergoing caesarean section….”*

Informal phrasing: Colloquial expressions (e.g., "almost a half") should be replaced with precise academic language.
**Author’s response**: Thank you! We have modified it accordingly.

Conclusion: The current conclusion is overly lengthy, reiterates results unnecessarily, and obscures the key takeaway. A more concise and impactful statement would be: "Policymakers and stakeholders should prioritize raising awareness about early prenatal ultrasound as a critical intervention for reducing maternal and perinatal morbidity and mortality."
**Author’s response**: Thank you very much for your suggestions. We have incorporated on the revised version. The revision reads as: “*Therefore, policy makers and other stakeholders should prioritize on availing prenatal ultrasound services at the health center or through early referral to hospital, creating awareness on the early prenatal ultrasound as a critical intervention for reducing maternal and perinatal morbidity and mortality. Furthermore, conducting large-scale research to identify the relationship between the frequency of ultrasound scans and its outcomes and addressing health facilities and care providers related factors are recommended.*”

Figure 1: A column chart would more effectively display proportions across gestational age categories than the current continuous line, which is better suited for time-series data.
**Author’s response**: Thank you very much. We accepted your comment and revised it accordingly.

Finally, the manuscript requires thorough stylistic revision, ideally by a native English speaker, as recurrent grammatical errors detract from its overall clarity and professionalism.

**Author’s response**: Thank you! We all authors have revised the article and it was also edited by a lecturer from Teaching an English as a foreign language. Furthermore, we have made language edition by using different online software. We hope it was improved in the revised version.

6. PLOS authors have the option to publish the peer review history of their article ([what does this mean?](https://track.editorialmanager.com/CL0/https:%2F%2Fjournals.plos.org%2Fplosone%2Fs%2Feditorial-and-peer-review-process%23loc-peer-review-history/1/010f0198661230ee-2bef11d0-52c5-4fc7-9fca-6d0abab0d947-000000/7yQ8_FIoB9rqPYoYAi2xyl18_gcIQBeAtH8S7o5ez0A=222)). If published, this will include your full peer review and any attached files.
If you choose “no”, your identity will remain anonymous but your review may still be made public.

**Do you want your identity to be public for this peer review?** For information about this choice, including consent withdrawal, please see our [Privacy Policy](https://track.editorialmanager.com/CL0/https:%2F%2Fwww.plos.org%2Fprivacy-policy/1/010f0198661230ee-2bef11d0-52c5-4fc7-9fca-6d0abab0d947-000000/qjvybNZXGPtC-i6_7TSdYHA9LY9jpURLTm_vkQBM6ss=222).

Reviewer #1: No

Reviewer #2: **Yes:** Dr Sailesh Bhattarai

Reviewer #3: No

Reviewer #4: No

Reviewer #5: No

Reviewer #6: **Yes:** Juan Carlos Bello-Muñoz

**Author’s response**: Dear reviewer we thank you for your time and valuable comments and suggestions.

Comments by Peninah Agaba

**Manuscript title: Timing of first prenatal Ultrasound and associated factors among women who gave birth at health institutions in Ambo Town, central Ethiopia.**

**Comments**

There are many typos and grammatical errors in the paper which should be corrected as soon as possible.

**Author’s response**: Dear reviewer we appreciate your comment and significantly improved in the revised version.

**Abstract**

Methods: Is the data source health facility records or exit interviews at the health facility or a mix of both. This should be made clear

**Author’s response**: It was a nice observation, thank you. We have revised it adding also record review as we used both.

**Results:** Why not say ‘Overall, 71%....

**Author’s response**: Thank you we have corrected accordingly.

**Conclusion:** women didn’t had is a wrong tense

**Author’s response**: Thank you! We have corrected it in the revised version. Now it reads as: “*In this study, more than a quarter of the women did not receive timely prenatal ultrasounds.”*

**Background:**

Paragraph 5, is the relationship between US and those factors confirmed or it could be due to other government campaigns. Use a word that shows the likely relationship

**Author’s response**: Thank you we have revised the paragraph accordingly.

Methods: in the abstract, you say participants were systematically selected while in the methods section, you say they were randomly selected. Which one is which?

**Author’s response**: Thank you! Yes, we used systematic random sampling technique to select study subjects. We mentioned randomly selected under the population section to show we used probability sampling.

The exclusion criteria is not right because this binary analysis should include yes and no. Include both in the analysis

**Author’s response**: Thank you for your concern. We excluded those women who did not undergone a prenatal ultrasound scan or who could not recall or had no record of their first ultrasound scan during their recent pregnancy were excluded. As our aim was to determine the timing of first ultrasound scan among those who had ultrasound scan.

How many days postpartum were these women? Were they interviewed from the ward or they had already been discharged and you found them at their homes?

**Author’s response**: We collected data at health facilities from postpartum women before they were discharged.

Attach a copy of the questionnaire

**Author’s response**: Thank you we will attach copy as an additional file along with the revised version.

**Sample size & sampling strategy**

The sample size determination write-up is not clear e.g what is reference 17 after 402 for? What is initial sample size? The word ‘of’ is missing in that sentence of initial sample size.

**Author’s response**: Thank you very much. We have revised it and now the paragraph read as: “*The sample size for the current study was determined using Epi Info statistical software version 7.1, employing the double population proportion formula. A 5% level of significance, an 80% power, and a 1:1 ratio of unexposed to exposed were considered. Using the variable "history of abortion," with 71.93% of outcomes in the exposed group and 58.13% in the unexposed group, along with an AOR of 1.845 (17), the sample size was calculated to be 402. After accounting for a 10% non-response rate, the final sample size was adjusted to 442.*”

The paragraph on sampling strategy is not clear. Present it in a clear & concise way. It is confusing the way it is. If you used records of July to September to get proportions, to guide the sample selection in each health facility, let it be clear. Which proportions were they that guided the samples in each health centres. Use percentages to give a picture of the proportions than absolute numbers- proportions of births & proportions for sample distribution.

The selection processes of participants must be described succinctly

**Author’s response**: Thank you very much. We have included the proportions of allocation for all health facilities. We added one figure of the schematic presentation for our sampling procedure to clearly depict the sampling procedure. The revised version now reads:

*“A systematic random sampling method was employed to recruit respondents. The performance report from July 23 to September 5, 2022, served as a reference for proportionally allocating study subjects to health institutions. A total of 758 postpartum women were identified at public health institutions in Ambo town. Specifically, 305 women were from Ambo University Referral Hospital, 341 from Ambo General Hospital, 45 from Ambo Health Centre, and 67 from Awaro Health Centre. Consequently, the sample was proportionally distributed across these selected health institutions: 178 (40.3%) participants were allocated to Ambo University Referral Hospital, 199 (45.0%) to Ambo General Hospital, 26 (5.9%) to Ambo Health Centre, and 39 (8.8%) to Awaro Health Centre. Finally, using the delivery registration book as a sampling frame, every second woman who gave birth was selected for the study, with the first participant chosen randomly (****Fig. 1).”***

I would like to know how this study becomes cross-sectional

**Author’s response**: As the current study mainly focused on determining the timing of first prenatal ultrasound and its associated factors at a single point in time over a short period of time. We didn’t categorized them based on their outcome or exposure status and therefore, we believe it is a cross sectional with analytical part in nature.

In the study area, you talked about several health facilities, how did you zero down on the three health facilities

**Author’s response**: Yes, it true that we described the available private and public health facilities in Ambo Town which are providing maternal health services such as Antenatal care. But only four public health facilities in the town are providing delivery services. Hence, we described them to show the context of the study setting in the town.

How many respondents for the pre-test? Were they different from the ones included in the final sample or you included pre-test data in the final analysis?

**Author’s response**: Thank you. We conducted our pre-test on 22 women and the data from the pre-test were not included in the final analysis.

Dis you use paper based questionnaires or computer assisted personal interviews?

**Author’s response**: we used paper based to collect data through an interview and records review.

What other independent variables were included in the model?

**Author’s response**: Thank you for your comment. We added one category “variables of the study” under the methods and materials section. Additionally, those variables which are fitted into the final model were indicated in the table.

*“In this study, the timing of the first prenatal ultrasound was used as the dependent variable, while a variety of independent variables were examined, categorized into several groups. Socio-demographic variables included the mother's age, place of residence, marital status, religion, ethnicity, educational attainment of both the mother and her husband, maternal occupation, and household income. Maternal factors considered were prior knowledge of timely prenatal ultrasound scans, previous experience with such scans, any health issues before or during pregnancy, whether the pregnancy was intended, alcohol consumption during pregnancy, and the presence of multiple pregnancies. Antenatal care (ANC) history variables encompassed the timing of the first ANC visit, the total number of ANC visits, and partner support during pregnancy. Reproductive history variables included gravidity, parity, history of abortion, history of congenital anomalies, and history of premature birth. Birth-related factors covered mode and place of delivery, history of Caesarean section, and gestational age at birth. Additionally, the study evaluated participants' knowledge and attitudes toward prenatal ultrasound.”*

**Results**

**Descriptive results**

The first sentence has a wrong tense- the word were is misplaced

On average income, remove brackets because the figures are part of the sentence otherwise, the sentence is incomplete

**Author’s response**: Thank you for your comment. We revised it accordingly to: “A total of 442 postpartum mothers participated in the study, resulting in a 100% response rate.” “Additionally, 352 (79.6%) of mothers resided in urban areas, and 247 (55.9%) reported an average monthly income ranging from 1000 to 5000 ETB”

‘Had an information’, ‘received from health professional’ these are not right, re-write them

Were delivered in the hospitals or delivered in the hospital

Were weighting????

**Author’s response**: Thank you for your suggestions. We appreciate your comment and modified it accordingly. The sentences now read:

*“Nearly half of the participants, 227 (51.4%), were informed about the ideal time for a prenatal ultrasound scan, and 167 (37.8%) of them received this information from a health professional.”*

*“Majority of the babies, 423 (95.7%) were born at term, with 404 (91.4%) weighing between 2500-4000g, and 46 (10.4%) were admitted to the Neonatal Intensive Care Unit.”*

**Factors associated with timing of first prenatal ultrasound**

To timely use ultra sound scan ot to have a timely ultra sound scan??

Check your brackets as you report the AOR and CIs

**Author’s response**: Thank you very much, we accepted your comment and revised it accordingly.

Could you briefly talk about the bivariate results? Mentioning them where you have is confusing as the reader expects multivariate factors & then you begin with bivariate factors.

I think the variable should be previous history of prenatal ultrasound

**Author’s response**: Thank you we accepted your comment and modified it accordingly.

The referencing style seems to be having issues, why do you have two brackets with different references in one place. You need to leave a space befor the brackets throughout the document

**Author’s response**: Thank you very much for your valuable comments. We have modified it in the whole document.

Overall, we are thankful for your constructive comments and suggestions which helped us to significantly improve our article.
