# Supplementary material for: Timing of first prenatal ultrasound and associated factors among women who gave birth at health institutions in Ambo Town, central Ethiopia
Source: PLoS One. 2025 Dec 5;20(12):e0338436. doi: 10.1371/journal.pone.0338436 (PMC12680356; doi:10.1371/journal.pone.0338436)
Supplement: Attachment — Submitted filename: R2 Response to Reviewers.docx [file pone.0338436.s005.docx]

**PONE-D-25-08139R1**

**Timing of first prenatal Ultrasound and associated factors among women who gave birth at health institutions in Ambo Town, central Ethiopia**

**PLOS ONE**

**To: Editor of Plos One Journal**

**Dear Sir/Madam,**

We sincerely express our gratitude for your efforts in reviewing our article and forwarding you valuable comments, and suggestions. We are also excited for considering our article further for publication. In accordance with the feedback provided by the reviewers and editor, we have revised and modified the manuscript, addressing each comment individually as detailed below. The revised version, with tracked changes and clean version are attached for your reference. The responses to the reviewers' and editor's comments are outlined below.

and considering our article for revision

**Gizachew Abdissa Bulto**

**Email:** [**gizachab@yahoo.com**](mailto:gizachab@yahoo.com)

**Corresponding Author**

**Editor Comment:**

Thank you for submitting your manuscript to PLOS ONE. After careful consideration, we feel that it has merit but does not fully meet PLOS ONE’s publication criteria as it currently stands. Therefore, we invite you to submit a revised version of the manuscript that addresses the points raised during the review process.
**Author’s response**: Thank you very much for considering our article for publication. We are grateful for your valuable comments, and suggestions.

Please submit your revised manuscript by Oct 23 2025 11:59PM. If you will need more time than this to complete your revisions, please reply to this message or contact the journal office at [plosone@plos.org](mailto:plosone@plos.org). When you're ready to submit your revision, log on to https://www.editorialmanager.com/pone/ and select the 'Submissions Needing Revision' folder to locate your manuscript file.
Please include the following items when submitting your revised manuscript:

- A rebuttal letter that responds to each point raised by the academic editor and reviewer(s). You should upload this letter as a separate file labeled 'Response to Reviewers'.
- A marked-up copy of your manuscript that highlights changes made to the original version. You should upload this as a separate file labeled 'Revised Manuscript with Track Changes'.
- An unmarked version of your revised paper without tracked changes. You should upload this as a separate file labeled 'Manuscript'.

If you would like to make changes to your financial disclosure, please include your updated statement in your cover letter. Guidelines for resubmitting your figure files are available below the reviewer comments at the end of this letter.

**Author’s response**: Thank you! We did it accordingly. We haven’t made change on our financial statement.

**Journal Requirements:**

If the reviewer comments include a recommendation to cite specific previously published works, please review and evaluate these publications to determine whether they are relevant and should be cited. There is no requirement to cite these works unless the editor has indicated otherwise.

**Author’s response**: Thank you! There is no such recommendation forwarded from the reviewer.

**Reviewers' comments:**
Reviewer's Responses to Questions

**Comments to the Author**
1. If the authors have adequately addressed your comments raised in a previous round of review and you feel that this manuscript is now acceptable for publication, you may indicate that here to bypass the “Comments to the Author” section, enter your conflict of interest statement in the “Confidential to Editor” section, and submit your "Accept" recommendation.

Reviewer #1: All comments have been addressed

Reviewer #2: (No Response)

Reviewer #4: All comments have been addressed

Reviewer #5: (No Response)

**Author’s response**: Thank you very much.

2. Is the manuscript technically sound, and do the data support the conclusions?

The manuscript must describe a technically sound piece of scientific research with data that supports the conclusions. Experiments must have been conducted rigorously, with appropriate controls, replication, and sample sizes. The conclusions must be drawn appropriately based on the data presented.

Reviewer #1: Yes

Reviewer #2: Yes

Reviewer #4: Partly

Reviewer #5: Partly

**Author’s response**: Thank you very much. We have addressed your comments further in the revision.

3. Has the statistical analysis been performed appropriately and rigorously?

Reviewer #1: Yes

Reviewer #2: Yes

Reviewer #4: Yes

Reviewer #5: Yes

**Author’s response**: Thank you very much.

4. Have the authors made all data underlying the findings in their manuscript fully available?
The [PLOS Data policy](http://track.editorialmanager.com/CL0/http:%2F%2Fwww.plosone.org%2Fstatic%2Fpolicies.action%23sharing/1/010f01992bfef095-76930546-4cf9-4a88-957b-aff08794a5cd-000000/Jj2tqb2NGj3KJUYdbdYJxHfSLW3WNIRFAZdWFLOlHDo=227) requires authors to make all data underlying the findings described in their manuscript fully available without restriction, with rare exception (please refer to the Data Availability Statement in the manuscript PDF file). The data should be provided as part of the manuscript or its supporting information, or deposited to a public repository. For example, in addition to summary statistics, the data points behind means, medians and variance measures should be available. If there are restrictions on publicly sharing data—e.g. participant privacy or use of data from a third party—those must be specified.

Reviewer #1: Yes

Reviewer #2: Yes

Reviewer #4: Yes

Reviewer #5: Yes

**Author’s response**: Thank you very much.

5. Is the manuscript presented in an intelligible fashion and written in standard English?
PLOS ONE does not copyedit accepted manuscripts, so the language in submitted articles must be clear, correct, and unambiguous. Any typographical or grammatical errors should be corrected at revision, so please note any specific errors here.

Reviewer #1: Yes

Reviewer #2: Yes

Reviewer #4: Yes

Reviewer #5: Yes

**Author’s response**: Thank you!

**6. Review Comments to the Author**
Please use the space provided to explain your answers to the questions above. You may also include additional comments for the author, including concerns about dual publication, research ethics, or publication ethics. (Please upload your review as an attachment if it exceeds 20,000 characters)

Reviewer #1: (No Response)

**Author’s response**: Thank you, we are grateful for your earlier comments.

Reviewer #2: Most of the comments regarding confounders have been addressed and are appropriately framed within the limitations section of this paper. highlighting a need for future research with more comprehensive data collection.
**Author’s response**: Thank you, your comments were invaluable for us to enrich our article.
Regarding the knowledge and attitude questions, what was the nature of options in each items? Yes, no, don't know or likert scale? Please clearly mention it what the total highest score would be in each construct.
Thank you.

**Author’s response**: Thank you very much. We have incorporated this in the revised version under the operational definition section. We have already added under the result section on page 10.

**The revision now read**:

*“Knowledge of prenatal ultrasound: Twelve questions with yes, no and I don’t responses were used to assess participants' knowledge of prenatal ultrasound. The average score was used to determine the status with the minimum and maximum scores of 0 to 12.” “The attitude score was determined based on a composite value derived from the responses to a 5-point Likert-type scale ranging from strongly agree to strongly disagree with the score ranging from 0 to maximum of 50.”*

Reviewer #4: 1) The discussion occasionally overstates causality (e.g., "good knowledge leads to timely scans”). Reframe findings as associations only.

**Author’s response**: Thank you. We have already stated in the first statement as maternal knowledge is associated with having timing of ultrasound scan. The second statement explains this as the association was twice among those who had overall good knowledge of prenatal ultrasound as compared to others.

2) Expand on health system factors (availability of machines, trained staff) that were not measured but could explain the stronger effect seen for hospital-based ANC.

**Author’s response**: Thank you, we share your concern too. We have acknowledged these under the limitation section of this article.

3) The authors may consider adding the implications of their findings for midwifery practice, for example, highlighting the role of midwives in providing antenatal education and making timely referrals to ensure that pregnant women receive an ultrasound before 24 weeks.

**Author’s response**: Thank you. We have accepted your comment and revised it accordingly. We added a statement which read:

*“These findings imply that, the health centers, obstetric care providers and policymakers at different level should prioritize and work on making prenatal ultrasound services accessible to all women at the health centers or through early referral to where the service is available. Moreover, awareness creation activities must also be considered for improving timely prenatal ultrasound scan.”*

Reviewer #5: I think most of my comments have been addressd. However I still feel that you should at least indicate the proportion of women who do not have any US at all. Otherwise it is a bit misleading to remark on the proportion of women who have timely US as this is not a proportion of the total of all pregnant women. You could say, "of the women who had an US scan, the proportion who ...etc" But even so it would add greatly if you could comment on the proportion of women who did or did not have any US

**Author’s response**: Thank you, we understand your concern, there are indeed women who do not had prenatal ultrasound during their current pregnancy. But, the current study was specifically focused on the timing of their first prenatal ultrasound among women who had at least one scan during their index pregnancy. While determining the proportion of women who never had an ultrasound would also be valuable for intervention, for us it would not be possible to analyze the timing for those without a scan along with those who had it. For this reason, we excluded those women with no ultrasound from the current study. The current study also didn’t accurately capture the number of women who had no prenatal ultrasound scan.

Dear Reviewers and editor, we are very much thankful for your constructive comments and suggestions which helped us to significantly improved our paper.

Thank you!
