# Supplementary material for: Timing of first prenatal ultrasound and associated factors among women who gave birth at health institutions in Ambo Town, central Ethiopia
Source: PLoS One. 2025 Dec 5;20(12):e0338436. doi: 10.1371/journal.pone.0338436 (PMC12680356; doi:10.1371/journal.pone.0338436)
Supplement: Attachment — Submitted filename: R3 Author response to reviewers.docx [file pone.0338436.s006.docx]

**PONE-D-25-08139R2**

**Timing of first prenatal Ultrasound and associated factors among women who gave birth at health institutions in Ambo Town, central Ethiopia**

**PLOS ONE**

**To: Editor of PLOS One Journal**

**Dear Sir/Madam,**

We sincerely express our gratitude for your efforts in reviewing our article and forwarding valuable comments and suggestions. We are also excited for considering our article further for publication. In accordance with the feedback provided by the reviewer and editor, we have revised the manuscript and addressed the reviewer comment individually below. The revised version, with tracked changes and clean manuscript are attached for your reference.

**Gizachew A Bulto**

**Email:** [**gizachab@yahoo.com**](mailto:gizachab@yahoo.com)

**Corresponding Author**

**Reviewers' comments:**

**Reviewer's Responses to Questions**

**Comments to the Author**

1. If the authors have adequately addressed your comments raised in a previous round of review and you feel that this manuscript is now acceptable for publication, you may indicate that here to bypass the “Comments to the Author” section, enter your conflict of interest statement in the “Confidential to Editor” section, and submit your "Accept" recommendation.

Reviewer #4: All comments have been addressed

Reviewer #5: (No Response)

**Author’s response**: Dear reviewers, we thank you very much!

2. Is the manuscript technically sound, and do the data support the conclusions?

The manuscript must describe a technically sound piece of scientific research with data that supports the conclusions. Experiments must have been conducted rigorously, with appropriate controls, replication, and sample sizes. The conclusions must be drawn appropriately based on the data presented.

Reviewer #4: Yes

Reviewer #5: No

**Author’s response**: We thank you for your comment we have addressed your concern below in detail.

3. Has the statistical analysis been performed appropriately and rigorously?

Reviewer #4: Yes

Reviewer #5: Yes

**Author’s response**: thank you very much!

4. Have the authors made all data underlying the findings in their manuscript fully available?

The PLOS Data policy requires authors to make all data underlying the findings described in their manuscript fully available without restriction, with rare exception (please refer to the Data Availability Statement in the manuscript PDF file). The data should be provided as part of the manuscript or its supporting information, or deposited to a public repository. For example, in addition to summary statistics, the data points behind means, medians and variance measures should be available. If there are restrictions on publicly sharing data—e.g. participant privacy or use of data from a third party—those must be specified.

Reviewer #4: Yes

Reviewer #5: Yes

**Author’s response**: Thank you!

5. Is the manuscript presented in an intelligible fashion and written in standard English?

PLOS ONE does not copyedit accepted manuscripts, so the language in submitted articles must be clear, correct, and unambiguous. Any typographical or grammatical errors should be corrected at revision, so please note any specific errors here.

Reviewer #4: Yes

Reviewer #5: Yes

**Author’s response**: We thank you, your comments have helped us to improve our article!

6. Review Comments to the Author

Please use the space provided to explain your answers to the questions above. You may also include additional comments for the author, including concerns about dual publication, research ethics, or publication ethics. (Please upload your review as an attachment if it exceeds 20,000 characters)

Reviewer #4: (No Response)

Reviewer #5: Thank you for your response but without addressing my comments the entire paper is quite misleading. You make claims about the proportion of women who have timely scans which is just not correct unless you include all the women who deliver at the facility, not just those who have a scan at some time or another. This remains unclear in your paper and as such I cannot recommend publication. Please include some data concerning the numbers of women who had no scan at all. You can compare this to the proportion of those who had a timely scan as compared to a late scan but you must state the total who could have had a scan but did not. Otherwise the claims in the papar as it is written are simply misleading.

**Author’s response**: Dear reviewer, we thank you very much for your thoughtful comment. We fully understand and appreciate the concern regarding the inclusion of women who had no ultrasound scans.

Our study, however, was specifically designed to assess the timing of the first prenatal ultrasound and its associated factors among women who gave birth at health institutions in Ambo Town and had at least one prenatal ultrasound. Since the primary outcome variable was the timing of the first scan, women who did not undergo any ultrasound were not eligible for inclusion, as their data would not contribute to the study’s objective.

To prevent any misunderstanding, we have now clarified this point in the manuscript as follows:

**Methods section under population on page 5 we revised the statements as:**

“All randomly selected women who delivered at public health facilities in Ambo Town and had at least one prenatal ultrasound scan were the study population. Women who did not undergo a prenatal ultrasound scan, could not recall, or had no record of their first ultrasound scan during their recent pregnancy were excluded.”

Finally, we have added a statement in the Limitations section to acknowledge that our findings do not represent the overall coverage of prenatal ultrasound use among all women, rather the timing of ultrasound scan among those who received at least one scan.

The revision now read as:

*“This study had the following main limitations. The study used a cross-sectional design, which makes it difficult to establish causality. Furthermore, as the study exclusively included women who underwent ultrasound scans and delivered at public health facilities in Ambo Town, the results may not be generalizable to women who gave birth at home or did not receive an ultrasound scan. Recall bias might be introduced for some variables that require women to remember past events to respond. The current study does not consider factors related to health facilities and care providers, which could be important to comprehensively explore the factors that could affect perinatal ultrasound scans and their timing.”*

We hope this clarification adds transparency and prevents potential misinterpretation of our results and addresses the reviewer’s concern to reflect the scope and purpose of our study.

7. PLOS authors have the option to publish the peer review history of their article (what does this mean?). If published, this will include your full peer review and any attached files.

If you choose “no”, your identity will remain anonymous but your review may still be made public.

Do you want your identity to be public for this peer review? For information about this choice, including consent withdrawal, please see our Privacy Policy.

Reviewer #4: No

Reviewer #5: No

We are thankful for your comments and suggestions.
